# High fat diet induces sex-specific differential gene expression in *Drosophila melanogaster*

**DOI:** 10.1371/journal.pone.0213474

**Published:** 2019-03-12

**Authors:** Tsering Stobdan, Debashis Sahoo, Priti Azad, Iain Hartley, Erilynn Heinrichsen, Dan Zhou, Gabriel G. Haddad

**Affiliations:** 1 Department of Pediatrics, Division of Respiratory Medicine, University of California San Diego, La Jolla, California, United States of America; 2 Department of Computer Science and Engineering, University of California San Diego, La Jolla, California, United States of America; 3 Department of Neurosciences, University of California San Diego, La Jolla, California, United States of America; 4 Rady Children's Hospital, San Diego, California, United States of America; Biomedical Sciences Research Center Alexander Fleming, GREECE

## Abstract

Currently about 2 billion adults globally are estimated to be overweight and ~13% of them are obese. High fat diet (HFD) is one of the major contributing factor to obesity, heart disease, diabetes and cancer. Recent findings on the role of HFD in inducing abnormalities in neurocognition and susceptibility to Alzheimer’s disease are highly intriguing. Since fundamental molecular pathways are often conserved across species, studies involving *Drosophila melanogaster* as a model organism can provide insight into the molecular mechanisms involving human disease. In order to study some of such mechanisms in the central nervous system as well in the rest of the body, we investigated the effect of HFD on the transcriptome in the heads and bodies of male and female flies kept on either HFD or regular diet (RD). Using comprehensive genomic analyses which include high-throughput transcriptome sequencing, pathway enrichment and gene network analyses, we found that HFD induces a number of responses that are sexually dimorphic in nature. There was a robust transcriptional response consisting of a downregulation of stress-related genes in the heads and glycoside hydrolase activity genes in the bodies of males. In the females, the HFD led to an increased transcriptional change in lipid metabolism. A strong correlation also existed between the *takeout* gene and hyperphagic behavior in both males and females. We conclude that a) HFD induces a differential transcriptional response between males and females, in heads and bodies and b) the non-dimorphic transcriptional response that we identified was associated with hyperphagia. Therefore, our data on the transcriptional responses in flies to HFD provides potentially relevant information to human conditions including obesity.

## Introduction

Diet consisting of high saturated fat (HFD) is a risk factor associated with cardiovascular disease (CVD) [1–3]. HFD is the most lethal habit after smoking [4] and a main contributing factor for obesity pandemic [5, 6]. A recent WHO report suggests that ~1.9 billion (39%) adults are overweight (http://www.who.int). These data suggest that obesity worldwide has tripled from 3·2% in 1975 to 11% in 2016 in men, and from 6·4% to 15% in women. Alarmingly, during the same period, childhood and adolescent obesity has increased tenfold in both girls (5 million to 50 million) and boys (6 million to 74 million) [7]. Globally, the United States is positioned at the top with an adult obesity prevalence rate between 38.3%-40.4% (+26.5% overweight) in women, 34.3%-37.7% (+38.7% overweight) in men [8, 9] and age-standardized childhood obesity at 12.7% [10]. At this rate, obesity will not only have a severe impact on associated chronic diseases like diabetes [11], cardiovascular disease [12], cancer [13], and osteoarthritis, but it is also expected to influence the basic socioeconomic development of entire countries [10].

Lately, HFD and obesity have also been identified as risk factors for neurological complications, both in the central and peripheral nervous system [14–22]. For example, HFD was found to accelerate cognitive decline [20, 22, 23]. It is also a known risk factor associated with Alzheimer’s disease [19], and anxiety and depression [24]. Animal studies have shown that HFD had a deleterious effect on the hippocampal-dependent memory [25] and together with stress it causes depression-like behavior [26]. Of note, recent studies have shown a sexually dimorphic facet in eating behavior [27] and the lower ability to suppress hunger in women [28] obesity susceptibility [29, 30], Alzheimer’s disease [19] and depression [31].

Previously we and others have shown that *Drosophila*, when fed HFD, showed increased triglyceride (TG) and glucose levels, decreased stress tolerance and lifespan, and activation of pathways involved in fat metabolism, insulin signaling, cardiac fat accumulation and dysfunction [32–34], quite similar to humans. There were other studies describing *Drosophila* as a model organism for human brain diseases including neurodegenerative diseases like Alzheimer’s disease [35, 36]. Additional features making *Drosophila* a useful organism include short life cycle, smaller but well-curated genome, easy to handle breeding and upkeep and, importantly, 75% of disease-related genes in humans have a *Drosophila* ortholog [37]. Hence, we hypothesized that a) HFD-induced transcriptomic changes in *Drosophila* could provide insight into the HFD- or obesity-related diseases affecting humans, including brain diseases and b) any sex-specific transcriptomic changes could provide relevant information for the sex-specific disparities in obesity or related diseases. In order to address the above hypothesis, we used in this study males and females *Drosophila*, fed on regular diet (RD) and HFD and analyzed the transcriptomic changes in the head and the rest of the body. We thus performed transcriptome-network-analysis that shed light on the HFD-related gender disparities.

## Materials and methods

### Regular and high-fat diet preparation

We used Jazz Mix Drosophila food from New Horizon Foods. For the regular diet (RD), as directed by New Horizon Foods, we added 800mL of distilled water to 151g food mix and heated to boil the mixture and placed 1mL each in regular size plastic vials. For the high-fat diet (HFD), as adopted in Heinrichsen et al., 2012, we added 20% weight per volume of food-grade coconut oil (Aunt Patty's Organic Coconut Oil) to the regular food [32, 34]. The hot food was stirred well until the oil is dissolved. Similar to the RD 1mL of medium was dispensed into each vial occasionally stirring the stock container.

### Fly rearing and collection

**Fig 1:**
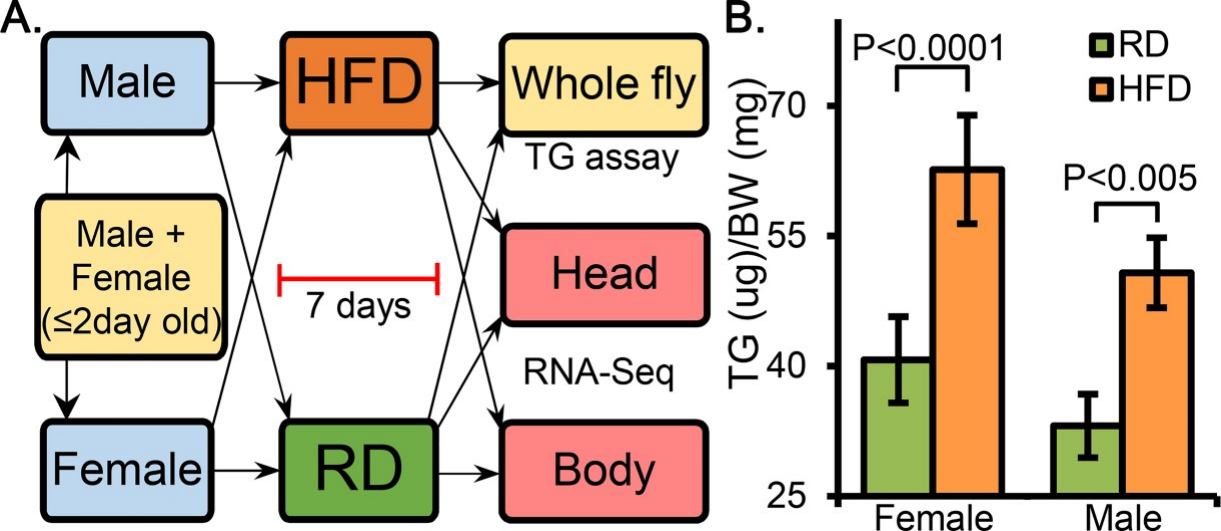
The protocol of HFD treatment and subsequent triglyceride (TG) levels. Schematic of high-fat diet (HFD) and regular diet (RD) treatment of the flies (A) and the TG levels measured after 1-week of treatment (B). Age matched flies were given the respective diets for 1 week. The TG levels were measured in triplicates in each group.

The *Drosophila* stocks *w1118* was obtained from Bloomington Stock Center. The flies were maintained on standard cornmeal diet and were kept in ambient condition of 25C with 12–12 light-dark cycle. Newly eclosed to 2 day old adult flies were transferred to a new vial containing standard cornmeal medium. After 24hrs the males and females were separated and each of them were further divided into experimental and control groups. The experimental groups of males and females were kept on the HFD and the control groups were kept on the RD. They were kept on respective diets for 7 days, in a room with 12–12 light-dark cycle, changing vials with respective diet on day 4. On day 7 the flies were used for different analysis. For the TG assay we used n = 7 males and n = 5 females, each in triplicates. For the transcriptome analysis, the flies were immediately frozen on dry ice and stored at -80C until use. A schematic diagram of the entire protocol is presented in Fig 1A.

### Triglyceride level measurement

After treating flies on RD and HFD for 7 days, a group of males (n = 7) and females (n = 5) flies were separately weighed and transferred to 2ml, 2.8mm ceramic bead tubes (MoBio). More males were used because of their smaller size. Each group was done in triplicates. Flies were then homogenized in 0.05% Tween using the Precelly's 24 homogenizer (bertin technologies), following which 500ul supernatant was transferred to an eppy tube. The eppy tubes were heated at 70° for 5 min (to inactive lipases) with gentle shaking, centrifuged for 3 min, 13.2k rpm, and only the supernatants were used for both TG and protein assays. TG levels were measured using the Infinity TG kit (Cat#. TR22421, Thermo Fisher Scientific) and protein levels were measured using normal BCA protein assay (Sigma–Aldrich, Saint Louis, MO).

### Tissue collection for RNA isolation

RNA was isolated in two different batches of flies at two different times. For the first batch, ~100 age-matched male or female flies kept for a week on RD and HFD were transferred to a 5ml polystyrene tube (BD bioscience) kept on liquid nitrogen. After a quick (~ 3x5second) vortex, the contents were poured onto mini-sieve column (Cat# F37845-1000, Scienceware), tapped few times so that the heads were filtered through the mini sieve and the bodies were retained. All the heads were then picked using a fine brush and transferred to 2ml, 1.4mm ceramic bead tubes (MoBio) containing trizole for RNA isolation vial. Simultaneously, we randomly picked 15 bodies separately (from the bodies that were retained on the mini sieve) and transferred to a different ceramic bead tubes (MoBio) containing trizole. RNA was isolated from both heads and bodies and was used for RNA-Seq analysis.

For RT-PCR validation a new batch of flies, both control and experimental, were collected and stored at -80C. Here the heads were manually removed from rest of the body. Both antennas were also separated from the heads. RNA was isolated from ~120 heads and 15 bodies and used for qRT-PCR validation.

### RNA-seq data generation and analysis

**Fig 2:**
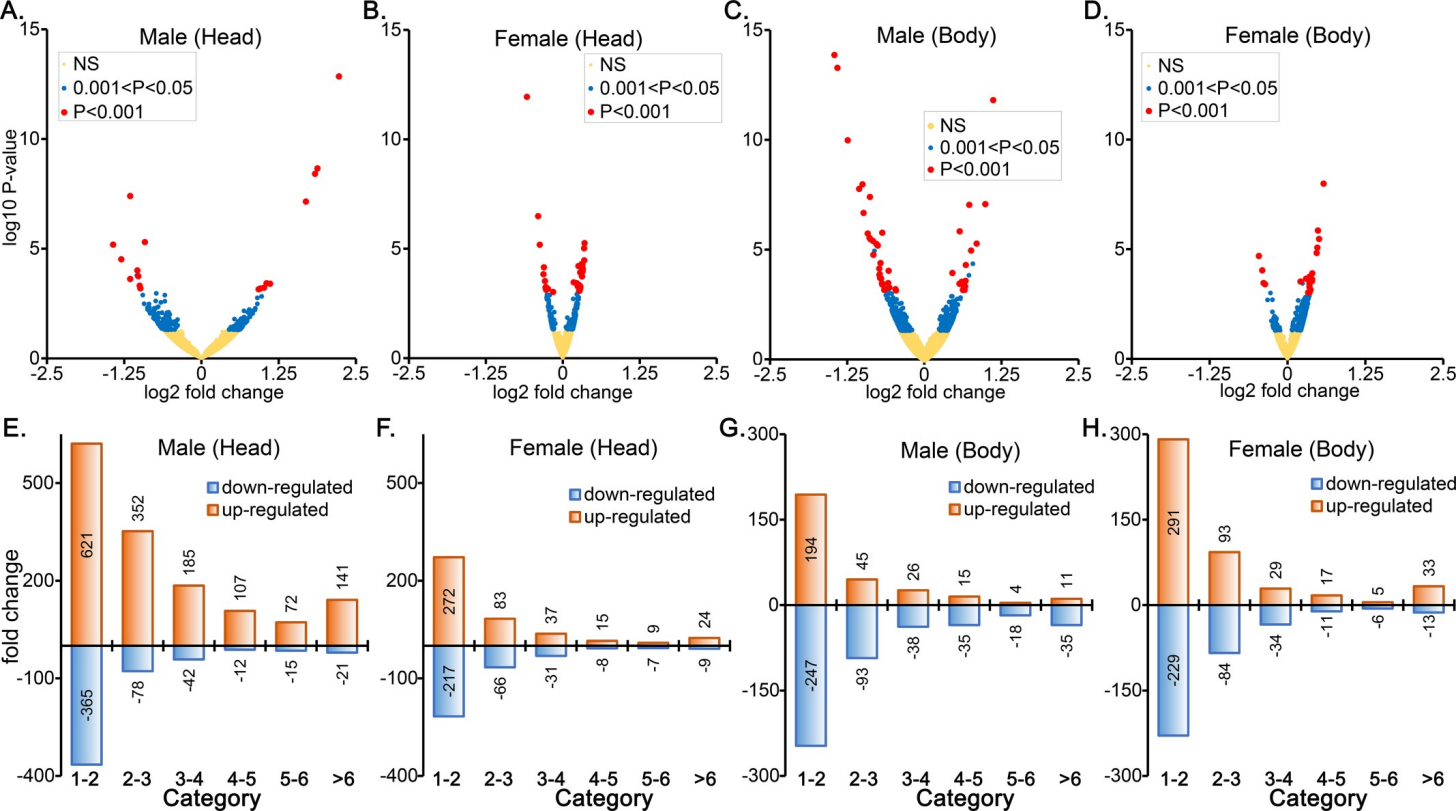
A robust transcription response to HFD in both heads and bodies of males than the females. The volcano plots depict log_10_ *P* values versus log_2_ fold change for genes in head (A and B) and body (C and D) of males (A and C) and female (B and D). The wider shape of volcano plot in the heads (A) and in bodies (C) of males indicates the males being more responsive to HFD (having wider distribution of fold change). *P*<0.001, red circle; 0.05<P<0.001, blue circle. The number of differentially expressed genes in different fold-change categories in the heads (E and F) and bodies (G and H) in the males (E and G) and female (F and H). Higher number of genes are upregulated than downregulated.

The RNA from the heads and bodies kept in RD and 7 days of HFD were submitted for RNA-seq. We used 500 ng of RNA with an RNA Integrity Number (RIN) of 8 or greater to generate libraries using Illumina’s TruSeq Stranded mRNA Sample Prep Kit (Illumina). Library preparation and RNA-seq were conducted at the IGM Genomics Center, University of California, San Diego. The manufacturer’s protocol was followed; with the exception that RNA was fragmented for 5 min. Libraries were multiplexed and sequenced with 100 base pair (bp) paired end (PE100) Rapid Run to a depth of approximately 300 million reads per sample on an illumina HiSeq2500. Sequences were aligned with Tophat by default settings for Drosophila melanogaster using the latest genome index as provided by Illumina (iGenome package). The aligned reads in BAM format were then used to determine differentially expressed genes between samples obtained from various conditions, using the mm9 GTF. The expression was statistically significant when adjusted P-value < 0.05. During the initial shortlisting, we considered Transcript Per Kilobase of exon per Million reads mapped (TPM) ≥ 1. The common genes that were differentially expressed (P<0.05) in different conditions were sorted (blue and red circles in Fig 2). The sensitivity of the technique enabled the detection of more transcripts, especially those that are of low-abundance. The expression, i.e., genes with TPM>0 in either the RD or HFD treated flies, was detected for 15,048 and 14,366 genes in male and female heads respectively and 15,467 and 13,900 genes in male and female bodies respectively (S1A and S1D Fig). The RNA-seq data related to our current findings are deposited to Gene Expression Omnibus (GEO), accession numbers GSE123240.

### RT-PCR validation

In order to further substantiate our results, we collected another batch of head RNA only, i.e., after removing antenna (as explained in RNA isolation). The antenna was separately collected (mentioned above). By doing an RT-PCR of antenna specific candidate genes, we observed that these differences were not statistically significant between HFD and RD. The expression in the antenna was more than 4x10^3^ fold of the head. For the candidate gene validation we picked the genes with P<0.01 and plotted the expression using heatmap. Within this list random genes with P<0.0001 were picked for RT-PCR validation.

### Gene ontology and network enrichment analysis

**Fig 3:**
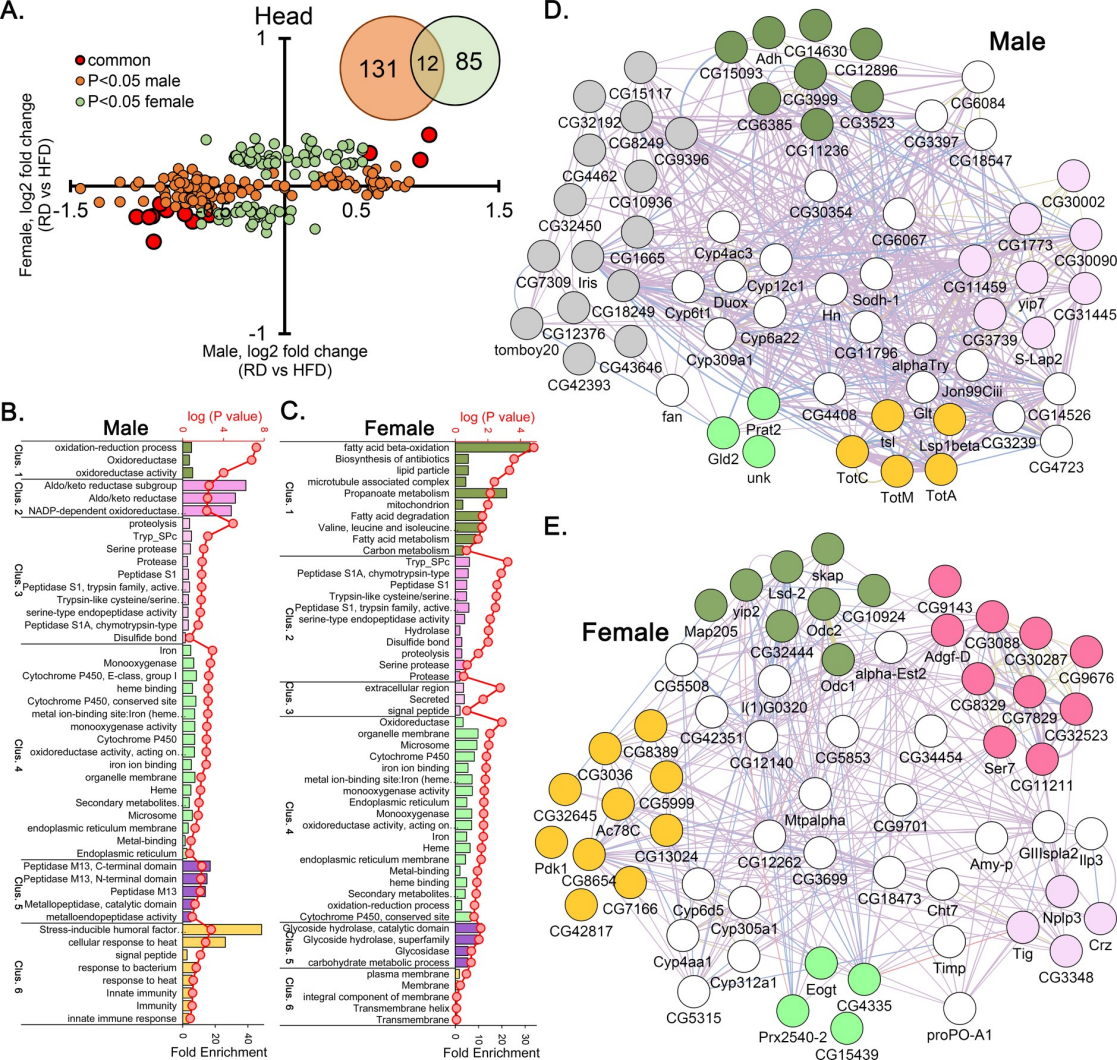
Transcriptional change in the heads leading to HFD-induced sex specific pathway enrichment. (A) Relative fold change between male and female heads after HFD treatment. Insert; Venn diagram depicts the number of differentially expressed genes shared between males and females head. Functional Annotation Clustering at medium stringency in the heads of male-specific (B) and female-specific (C) genes. (D) Network analysis depicting the individual genes within each cluster interacting in both within as well as between clusters for male (D) and female (E). Similar color coding for bar and network plots. White circles in network plots are genes shared between two or more clusters.

**Fig 4:**
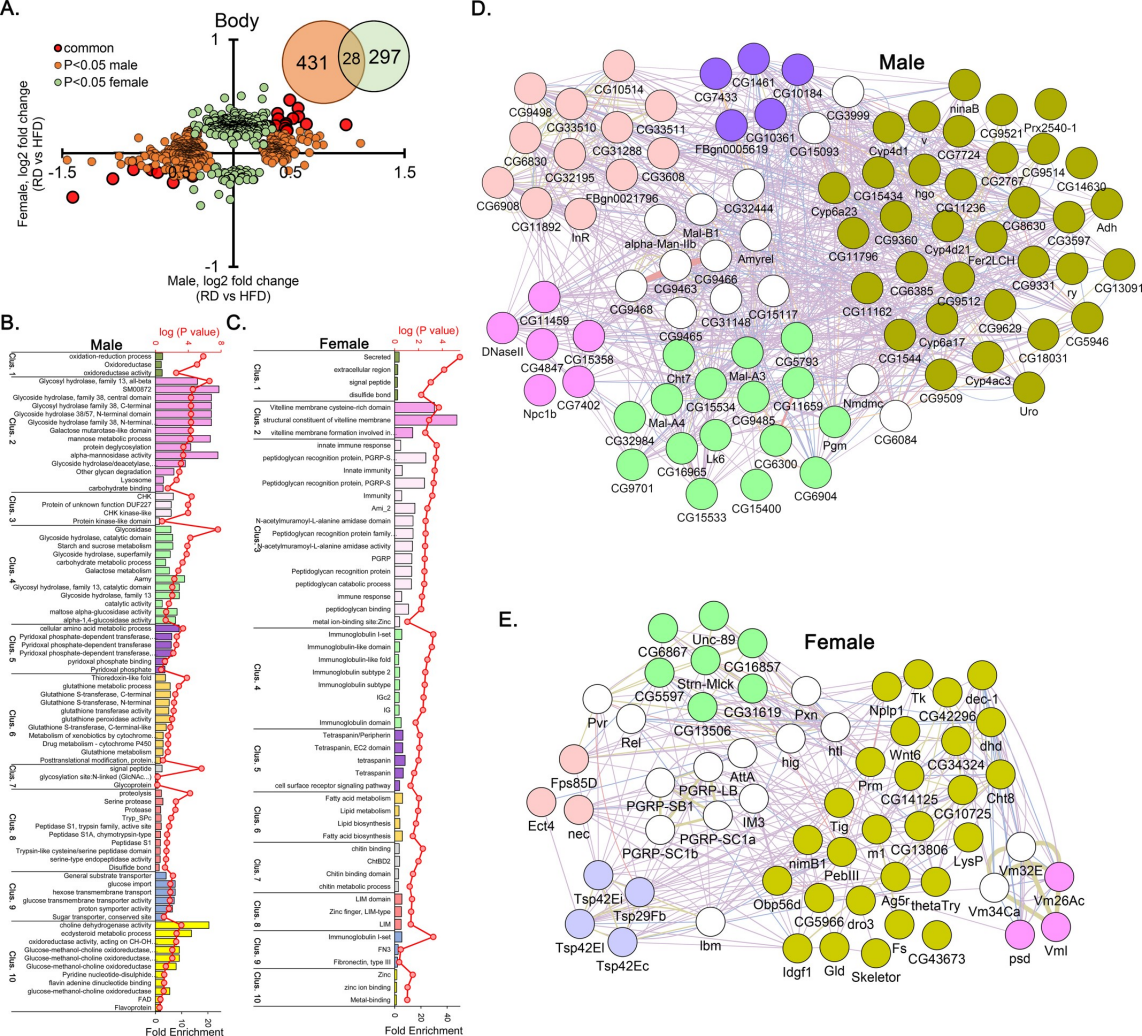
Transcriptional change in the bodies leading to HFD-induced sex specific pathway enrichment. (A) Relative fold change between males and females bodies after HFD treatment. Insert; Venn diagram depicts the number of differentially expressed genes shared. (B) The top 10 Functional Annotation Clusters at medium stringency in males (B) and females (C) body genes. Network analysis depicting the differentially expressed genes interacting within the clusters as well as between the clusters for males (D) and females (E) body genes.

To understand the molecular significance from the list of differentially expressed genes, we performed the batch annotation and gene-GO (gene ontology) term enrichment analysis using DAVID (http://david.niaid.nih.gov). For this analysis, we first categorized the differentially expressed genes into six different groups, 3 for head and 3 for bodies, i) genes common between males and females and maintained similar directionality of the fold-change (red circles in Fig 3A for heads and Fig 4A for bodies), ii) Male-specific (orange circles in Figs 3A and 4A) and iii) female-specific green circles in Figs 3A and 4A). Analysis for heads and bodies were carried out separately. The list of the genes was uploaded to DAVID using flybase gene ID as the identifier and the output was obtained using a functional analysis tool. The output was obtained in both ‘functional annotation chart’ and ‘functional annotation cluster’. In the ‘functional annotation chart’ all the GO terms were simply ranked based on their statistical significance i.e., P-values. Additionally, we used the default threshold settings of ≥2 GO category of final group membership that were compounded to detect overexpressed pathways into ‘functional annotation clusters’. These clusters were ranked based on their enrichment score. The GO terms not clustering were labelled as orphan GO categories. They may be important in spite of being a single entity.

The various types of interactions between the enriched genes are depicted using genemania (http://genemania.org) on Cytospace program (v3.4.0). We first identified the cluster membership for each of our gene. For body networks we only picked the genes belonging to the top 5 clusters because there were 36 and 26 cluster in males and females respectively. The networks depict interactions of both within a cluster and between clusters.

### Feeding assay

Groups of five flies kept on RD or HFD for 7 days were transferred onto fresh food medium containing 2.5% (w/v) blue dye (F. D. & C Blue Dye no. 1) mixed in RD or HFD. The flies were transferred to empty tubes after 30, 45 and 60 minutes of keeping on blue dye food (RD and HFD). At least three biological replicates were collected for each time point. After every time point the tubes were immediately snap frozen in liquid nitrogen and subsequently stored in -80C.

For quantification of food intake five flies for each replicate were homogenized in 200 μL of 1xPBS. A further 800 μL of distilled water was added and the suspension passed through a 0.22 μm Millex filter (Millipore Corporation, Bedford) to remove debris and lipids. The absorbance of the liquid sample was then measured at 629 nm [Hitachi U-2001 Spectrophotometer (Lambda Advanced Technology Ltd., UK)]. Age-matched flies exposed to non-dyed food were used as the baseline during spectrophotometry. The amount of labelled food in the fly was calculated from a standard curve made by serial dilution in water of a sample of blue food.

### Statistics

Results are presented as means ± standard deviations. Data comparison between groups was analyzed using two-way analysis of variance (ANOVA). All statistics were calculated using Analyse-it for Microsoft excel 4.18.1 (Analyse-it Software, Ltd.). Changes were considered statistically significant if p<0.05.

## Results

### Triglyceride levels in flies fed on a high-fat diet

We have previously evaluated the effect of a HFD consisting of 20% w/v of coconut oil (rich in saturated fats) in female Drosophila [32, 33]. A schematic diagram of the entire protocol is presented in Fig 1A. We found that female *w1118* flies kept on HFD showed a significant increase in whole-body TG levels when compared to flies kept on RD (*P*<0.0001). In our current studies, we measured whole-body TG levels in both males and females and as anticipated, both male and female flies showed significant increases in whole-body TG levels (*P*<0.005) when compared to their RD fed counterparts (Fig 1B).

### Robust transcriptional response to HFD in male flies

Our transcriptome comparisons of HFD and RD revealed a more robust transcriptional response in the males than the females, with a higher number of differentially expressed genes both in the heads and in the bodies (Fig 2). For example, the overall head comparisons, revealed 143 genes in the males and only 93 in the females heads (Fig 2A and 2B, blue and red circles combined) that differed significantly (*P*<0.05). Similarly, at an FDR adjusted *P* values of <0.05, the number of differentially expressed genes in the bodies were 464 in the males and 330 in the females (Fig 2C and 2D, blue and red circles combined). The results can also be viewed through the shape of volcano plots where males have a wider distribution of fold-change signals (broader ‘v’ shape) than the females for both heads and bodies (Fig 2A–2D). Additionally, the transcriptome analysis also revealed that in the heads more genes are upregulated than downregulated (Fig 2E and 2F), especially in the males. However, in the bodies, more genes were downregulated in the males and opposite in the females (Fig 2G and 2H). Overall, these results indicate that male flies when kept in HFD are more responsive or sensitive to the HFD. Interestingly, only four genes i.e., *Lsp2*, *Gnmt*, *Cyp4e3* and *CG5953*, were common in all the comparisons.

### Sex specific differential gene expression in the heads

In order to understand the effect of HFD on heads, we first identified the HFD-induced differentially expressed genes in the heads of males and females (FDR adjusted *P*<0.05). Considering the complexity of HFD-related phenotype and the reasoning that when groups of genes are analyzed together they reflect a potentially higher biological relevance, we chose to perform pathway enrichment analysis. We therefore categorized the differentially expressed genes into three different groups. Group-1 consisted of common genes statistically significant in both males and females and maintaining a similar directionality of the fold-change (n = 12, red highlighted in Fig 3A). Group-2 consisted of male-specific genes (orange highlighted in Fig 3A) and group-3 female-specific (green highlighted in Fig 3A). There were 131 and 85 genes in group-2 and 3 respectively (Venn diagram Fig 3A).

Analysis of group-1 genes, revealed a single cluster, the ‘oxidation-reduction process’ at ES>2. Group-2 genes revealed seven different clusters (Fig 3B). The ‘oxidation-reduction process’ cluster was again at the top. Cluster 2, which was 30–40 fold enriched was related to an aldo/keto reductase activity and was unique to group-2. This cluster consisted of only 3 genes, *CG18547*, *CG3397* and *CG6084* and is a subset of cluster 1 (Fig 3D). Cluster 3 (related to protease activity) and cluster 4 (related to cytochrome P450, heme and monooxygenase activity) were shared with group-3’s cluster analysis. Cluster 6 which was unique to males, represented GO terms related to stress responses (Fig 3B). This cluster consists of eight genes, all downregulated on HFD and was related to immune and stress responses, e.g., response to heat and UV (Fig 3D). This includes *TotA* and *TotC* genes from *Turandot* family reported to have roles in stress response. When we looked at the cluster analysis of group-3 genes i.e., female head-specific it had six enriched clusters (Fig 3C), and, as we mentioned, three were shared with group-2. Interestingly, the two group-3 (female head) specific clusters, cluster 1 and 5 were related to lipid and carbohydrate metabolism or glycosidase activity. The top cluster consisted of 17 genes (8 exclusive, Fig 3E) where 85% genes were upregulated. The genes that were common in all the GO categories in cluster 1, including fatty acid metabolism, were *Mtpalpha*, *yip2* and *CG12262*, and they were all upregulated in HFD treated female heads.

Additionally, some of the orphan GO categories (not part of any cluster) were also interesting to explore. For example, at the *P* <0.005, aromatic amino acid family metabolic process (GO:0009072), tyrosine metabolism (dme00350) and phenylalanine metabolism (dme00360) were individual GO categories enriched in the males. GO term related to sleep (GO:0030431) was the only enriched category at *P*<0.005 in the females. A detailed list of all the GO terms enriched is provided in S1 Table.

There were genes that were shared between different clusters and multiple pathways. The cytochrome P450 family genes (*Cyp4ac3*, *Cyp6t1*, *Cyp6a22*, *Cyp309a1* and *Cyp12c1*) are a perfect example as these genes were shared between cluster 1, 4 and 7 in the group-2 gene analysis (Fig 3D). Another important gene *Cyp4e3* from group-1 list i.e., upregulated in the heads of both genders, also from the same family. Together, they contributed to closely related GO terms e.g., oxidoreductase activity, highly enriched in different clusters. Another example is the shared genes in female heads that gave functionally related GO terms like fatty acid beta-oxidation (GO:0006635) in cluster 1 and oxidation-reduction process (GO:0055114) in cluster 4 (Fig 3E).

Overall, head transcriptome analysis revealed i) downregulation of genes related to stress response (cluster 6) in the males, ii) enriched pathways that were directly associated with fatty acid metabolism in the females (cluster 1) and iii) there were important genes, for example genes from cytochrome P450 family, that belonged to multiple biological processes.

### Augmented glycoside hydrolases activity in the male body and immune system in female body after High-fat diet

From the transcriptome analysis of bodies we identified 28 common (group-4), 431 male specific (group-5) and 297 female specific (group-6) differentially expressed genes (Fig 4A). Group-4 genes showed a GO cluster representing lipid catabolic process (ES = 2.35). From group-5 and group-6 we had 36 and 26 clusters respectively where the majority of clusters were sex specific. For example 7 of the top 10 clusters were sex-specific (Fig 4B and 4C). Interestingly, group-5 genes had significant overrepresentation of GO terms related to carbohydrate metabolism (Fig 4B). This included glycoside hydrolases in cluster 2 and cluster 4 that are directly related to oligosaccharides synthesis. There were other indirectly linked GO terms like pyridoxal phosphate activity in cluster 5, glutathione transferase activity in cluster 6, glucose transporters in cluster 9 and glucose-methanol-choline oxidoreductase in cluster 10 (Fig 4B).

We explored the gene networks in the top five clusters. The top male body cluster had 38 genes and was related to oxidoreductase (ES = 4.48, Fig 4D). In the female body, the top cluster was assigned 39 genes (ES = 3.72) and consisted of GO terms like signal peptide, extracellular region (GO:0005576), secreted (Fig 4E). Interestingly, numerous genes from this cluster were shared between clusters 3 and 4, both related to immune system.

The above analysis revealed an enrichment of oligosaccharides synthesis in the bodies of male flies kept on HFD and immune system related GO enrichment in the bodies of female flies. The female specific cluster 2 contains genes e.g., *Vm32E*, *Vml* and *Vm26Ac*, related to vitelline membrane formation involved in chorion-containing eggshell formation. It is also interesting to note that when analyzing the sex specific genes in both heads and bodies, only females showed GO term enriched for lipid metabolism. In contrast, these GO terms were absent in the heads and were low-ranking in the body of males (S2 Table).

### Identification of novel candidate gene/s differentially expressed to HFD

**Fig 5:**
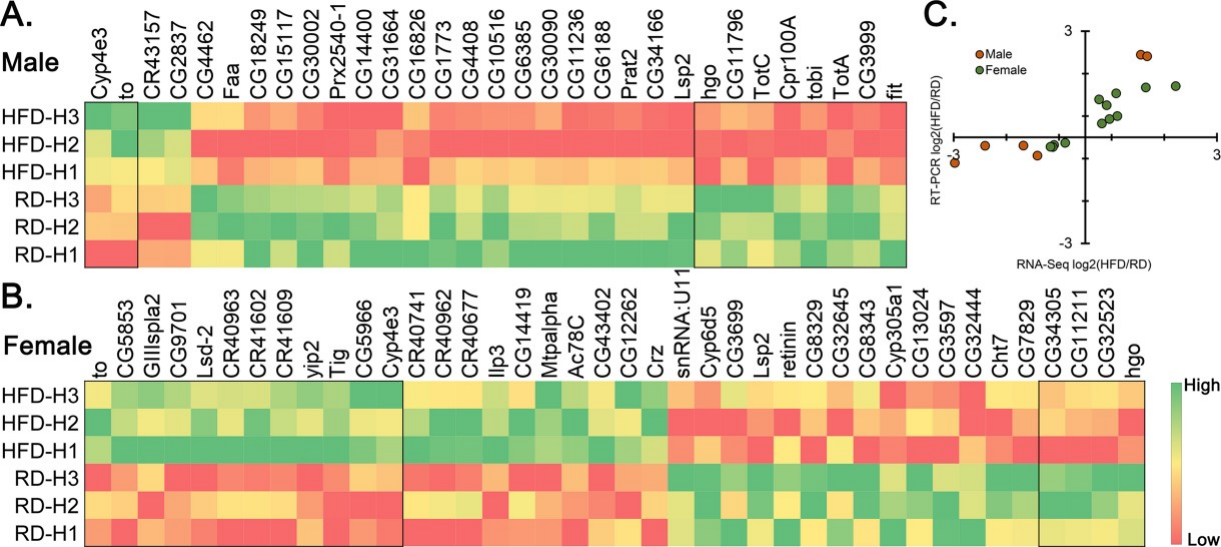
Heat map of differentially expressed genes in the heads. Heat map depicting differentially expressed genes in the heads of male (A) and female (B) based on RNA-seq results. The genes with P<0.001 are in box. (C) Correlation between RNA-Seq (X-axis) and RT-PCR (Y-axis) results from the HFD/RD expression change (S3 Table). Green color, upregulation; red, downregulation.

**Fig 6:**
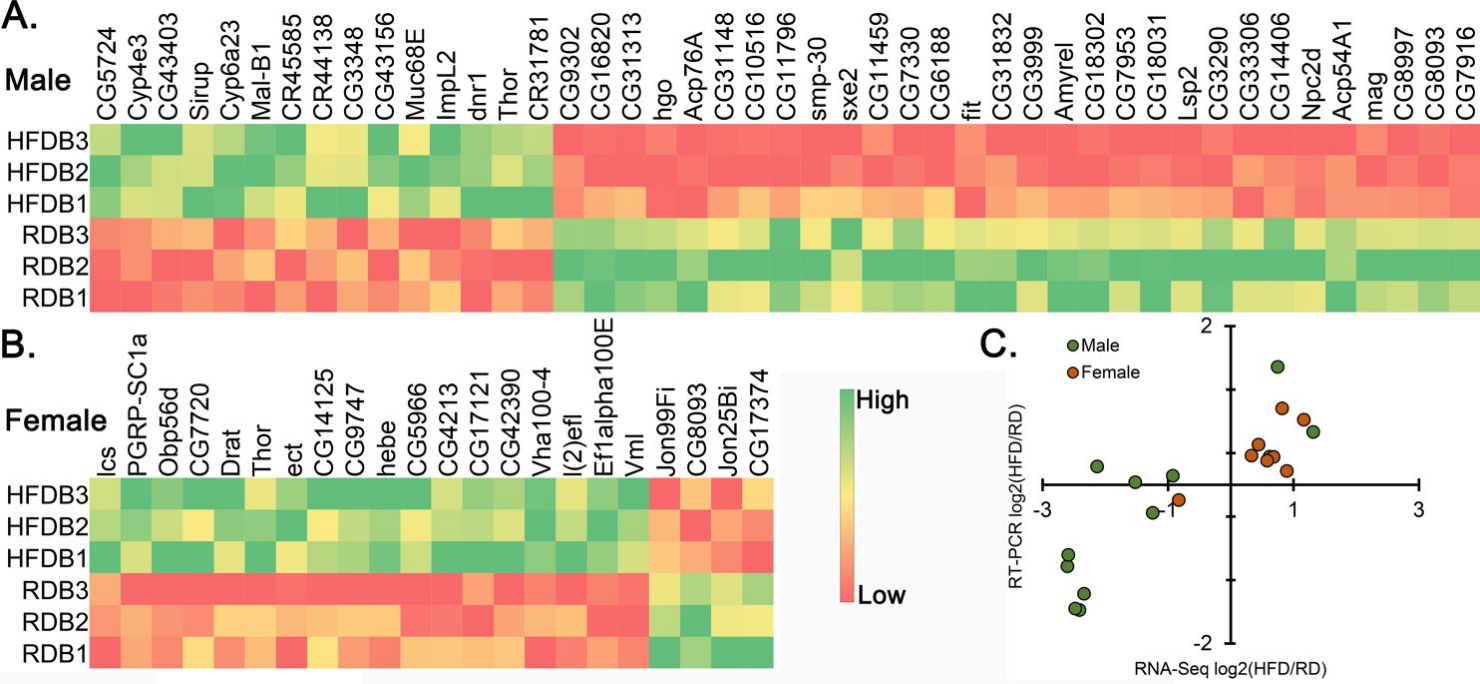
Heat map of differentially expressed genes in the bodies. Heat map of differentially expressed genes in the males (A) and female (B) body based on of RNA-seq (TPM values). The gene with P<0.001 in box. (C) Relative ration of HFD vs RD in the RNA-Seq (X-axis) compared to RT-PCR (Y-axis) results (S3 Table). Green color, upregulation; red, downregulation.

To investigate the differential expression further, we sorted the genes based on their p values. At *p*<0.001 there were 10 HFD-induced differentially expressed genes in male heads; there was an additional 21 genes at *p*<0.01 (Fig 5A). In the female head, 16 genes were differentially expressed at *p*<0.001 and an additional 24 genes at *P*<0.01 (Fig 5B). A clear separation between the HFD and RD can be seen in the heatmap plot (Figs 5 and 6). Unlike male heads where most of the genes were downregulated (27 down and 4 up), female heads showed almost an equal number of genes that were differentially expressed (22 up and 18 down). Only 4 genes, i.e., *Cyp4e3*, *takeout*, *Lsp2*, and *hgo*, were top-ranked and common between male and female heads. Interestingly, *Cyp4e3* was also significant in the bodies of males and females with a higher fold change in male body.

To make sure that the differential expression was head-specific, we isolated RNA from another batch of head samples, i.e., after removing the antennae. We then randomly picked a few top ranking genes for RT-PCR validation. All genes would either show statistically significant change in the RT-PCR or were similar in directionality of expression between the RT-PCR and the RNA-seq results (Fig 5C).

In the male body, there were 45 top ranked differentially expressed genes at P<0.001 (Fig 6A) and an additional 85 at P<0.01 (S2 Fig) where most of the genes were downregulated (84 down and 46 up). The female body had 22 genes at P<0.001 (Fig 6B) and additional 70 at P<0.01 (S2 Fig). Out of 92 genes only 8 genes were downregulated. Only nine genes, *CG8093*, *dnr1*, *CG34220*, *Vha100-4*, *Drat*, *CR31781*, *Odc1*, *CG15818* and *Thor* were common between the male and female body. To confirm the expression pattern of these genes we used RNA isolated from a new batch of body samples and performed RT-PCR on few randomly picked top ranking genes. A similar trend is maintained in almost all of the genes tested (Fig 6C).

### HFD induces hyperphagia in males and females flies

**Fig 7:**
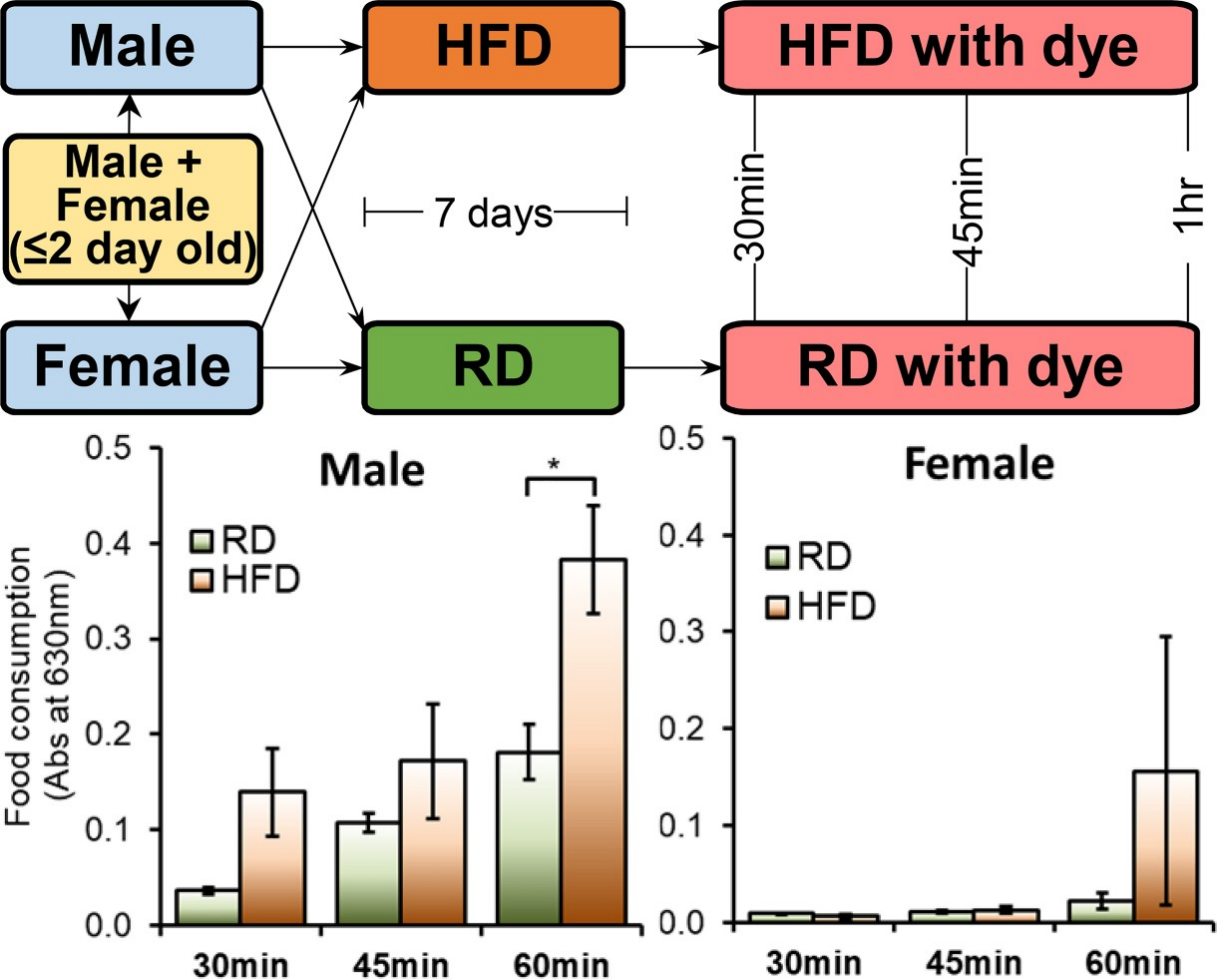
High fat diet induced hyperphagia in both males and females. (A) Schematic of the quantification of food consumption. The amount of food consumed by (B) males and (C) females that were chronically fed on a RD and a HFD. Food intake was measured at indicated time points.

One of the top candidate genes in our analysis was ‘*takeout*’ (also known as *to*), a gene which was reported to have a role in feeding behavior. This prompted an assessment on differences in food consumption in the flies kept on HFD. We did a controlled experiment to quantify the amount of food consumed at different time points i.e., 30, 45 and 60mins, in both males and females kept on RD and HFD (schematic provided in Fig 7A). Overall, it appeared that males were eating more food irrespective of the diet (Fig 7B and 7C). The HFD flies consumed more food than the RD flies. The difference in the males were visible early on at 30mins (Fig 7B) while in the females it appears only at 60mins (Fig 7C).

## Discussion

In the current study, we demonstrate that consumption of a HFD leads to diverse gene expression patterns in the heads and bodies of *D*. *melanogaster*. To our knowledge, this is the first comprehensive study that identified transcriptome changes in the heads and bodies of both males and females under normal condition and after the consumption of a HFD. Male and female comparisons of the body’s transcriptome are consistent with previous studies where a large number of genes (74.4%) are differentially expressed [38]. Since the comparisons of differentially expressed genes in the heads of male and female flies at the basal level itself depicts a large number of genes (e.g., 3240 genes, p<0.05), our focus here is to decipher the effect of HFD on the head and on the body of males and females respectively.

### Alteration in fatty acid metabolism in female flies

A female-centric enrichment of gene expression in lipid metabolism both in heads and bodies were very interesting (Figs 3E and 4E). The core genes, i.e., common in all the GO categories of cluster 1, in HFD-treated female heads including *Mtpalpha* (Mitochondrial trifunctional protein (MTP) α subunit), *yip2* (yippee interacting protein 2) and *CG12262* (Medium-chain acyl-CoA dehydrogenase), all are upregulated (Fig 5B) only in the female heads and are involved in mitochondrial β-oxidation. For example, *Mtpalpha* catalyzes long-chain fatty acid β-oxidation [39], *yip2* is a target gene of hepatocyte nuclear factor 4 (*Hnf4*) involved in lipolysis [40] and the role of *CG12262* in medium-chain-acyl-CoA dehydrogenase activity is inferred from its sequence similarity with *ACADM* (Acyl-CoA Dehydrogenase Medium Chain). As such, the baseline expression itself in female flies are high; it is further increased by the HFD treatment in the heads, which is intriguing. In contrast, in the males transcriptome analysis, the GO terms related to lipid metabolism were absent in the heads (Fig 3D). We have previously noted that female HFD-fed flies have a significantly altered fatty acid, amino acid, and carbohydrate metabolism [33]. The current study further depicts an efficient β-oxidation in the female heads.

To our knowledge, this is the first study reporting HFD-induced sex disparities at the transcriptomic levels in the heads. Although there are reports showing sex differences in susceptibility to weight gain in higher animals, e.g., mice and humans [41–44], it will be speculative at this stage to have any correlation. However, future studies can use our current findings to prioritize candidate genes.

### Decreased expression of stress response genes in males

Our transcriptomic analysis reveals HFD-induced male-specific enrichment of stress and innate immune response related GO terms. We found that all the genes in this cluster were significantly downregulated in the male heads but were unaffected in females. This includes *Turandot* genes, e.g., *TotA*, *TotC*, *TotM*, which are usually induced in response to a variety of stresses, including heat, mechanical stress, ROS, and bacteria [45]. In our previous study, we found that HFD in female flies had a detrimental effect on lifespan and stress tolerance, e.g., anoxia, cold and starvation [32] while the effect on male flies related to these phenotypes were not known. The male specific downregulation of *Turandot* genes is intriguing because this was present in only two other instances, one in DDT resistant strains [46] and the other in flies with overexpression in longevity genes [47]. Therefore, we believe that our current transcriptomic analysis provides data on the HFD-induced gender difference in stress response.

### Attenuated glycoside hydrolases activity in the male body

We also observed a male-specific glycoside hydrolase enrichment in our current study (cluster 2 and cluster 4, Fig 4B) where the genes of these clusters were significantly downregulated. The glucoside hydrolases are an important group of enzymes involved in carbohydrate metabolism [48]. One interesting viewpoint that emerged from this analysis is related to male gametogenesis. Gamete binding, i.e., sperm-egg interaction, is a carbohydrate-dependent event and glycosidase are also reported to be present on the surface of fly spermatozoa [49]. The candidate genes identified in this current study that are related to glycosidase activity may directly influence sperm quality and function, as the dietary fat is known to impair spermatogenesis [50] and lead to infertility in male obesity [51]. For example, subgroup of Lysosomal α-mannosidase genes *LManIII*, *LManIV*, *LManV* and *LManVI* (part of cluster 2 and 4) were all downregulated in the HFD males. Therefore, lower levels of these enzymes post HFD treatment indicates scarce enzyme-substrate complex formation involved in sperm-egg interaction in Drosophila [49].

### Differential expression of fat body specific genes

In the adults, a vast majority of lipids are stored in the adult fat body and are mobilized or accumulated to maintain a supply and demand equilibrium [52]. When fed a HFD, flies accumulate excess fat in both fat body and non-adipose tissue [34]. Therefore, the fat body is an important tissue when exploring HFD responses in *Drosophila*. We noted that only four genes were common under all the HFD conditions, i.e., differentially expressed in the head and body of males and females. These were *Lsp2* (larval serum protein 2) and *Gnmt* (Glycine N-methyltransferase) downregulated and *Cyp4e3* (Cytochrome P450 4e3) and *CG5953* are both upregulated. *Lsp2* gene is specifically expressed in the fat body of larvae and in the head’s fat body of the adult flies [53]. Transcription of Lsp2 is induced by steroid hormone, 20-hydroxyecdysone (20E), during third instar larvae that elicits metamorphosis [54]. Sparse reports in the adult flies indicate Lsp2’s role in activation of yolk protein (YP) genes both in female and male flies [55, 56]. Interestingly, despite being the reporter gene of the fat body, especially in the adult, its role in fat metabolism is lacking. We believe that the downregulation could be due to reduced lipolysis under high fat condition. This is supported by the fact that 20E, which stimulate Lsp2, is known to induce fat body lipolysis [57]. On the contrary, its expression is reportedly increased to >3 fold in a fly model with defective ability to breakdown dietary lipid i.e., when there is low TG in the fat body [58]. *Gnmt* is yet another gene in the heads of *Drosophila* that is fat-body specific [59, 60]. Interestingly, loss of *Gnmt* resulted in TG reduction indicating its important role in maintaining the proper levels of TG [60]. In an unrelated study, this gene was reportedly upregulated in the male heads post mating [59]. C*yp4e3* gene expression was also high in the adult fat body along with Malpighian tubules [61]. Its role in lipid accumulation is discussed here. The function of fourth gene *CG5953* is not known and future studies can focus on its role in lipid metabolism. The *takeout* is also a fat body-specific gene and is upregulated in both male and female heads post HFD treatment. Interestingly we saw an inverse correlation between *takeout* and *Lsp2* fold change. There are other instances where these two genes behave in a similar way [62].

### Correlation between lipid accumulation and *Cyp4e3* upregulation

Apart from the sex-specific differences, we also report here few fundamental HFD responses that are unrelatedly to sex or tissue specificity. We observed that *Cyp4e3* of cytochrome P450 family (CYPs) was among the top up-regulated genes in the heads (P<0.0001) and bodies (P<0.05) of both males and females in HFD. CYPs belong to a large family of diverse NADPH-dependent mono-oxygenases, conserved from bacteria to human that catalyzes chemical reactions involved in detoxification of foreign compounds and metabolism of endogenous compounds, including fatty acids. In *Drosophila*, *Cyp4e3* is among a few genes that were expressed in all three key metabolic tissues i.e., fat body, midgut and malpighian tubules, of feeding but not wandering third instar larvae [63]. Functionally, *Cyp4e3* plays an important role in regulating antioxidant function. The expression significantly increases on exposure to ethanol, H_2_O_2_, paraquat or insecticides [61, 64] or high sugar diet [65].

Interestingly, previous studies had not discovered a direct relationship between lipid accumulation and *Cyp4e3* upregulation. Whether it is ethanol exposure [64], high-sugar-fed larvae [65, 66] or exposure to permethrin [61, 67], all conditions show lipid accumulation and *Cyp4e3* upregulation but have overlooked this important relationship. This important observation was further supported by evidence in *Cyp4v3* (mouse ortholog) knockout mice (*Cyp4v3*^*−/−*^) and patients with mutations in *CYP4V2* (human ortholog) where a systemic dyslipidemia and a decreased metabolism of labeled fatty acid were detected [68]. And finally the formation of fused lipid droplet, fat storing cell organelles, in *Caenorhabditis elegans* ortholog *cyp-37A1* mutants [69] clearly illustrates our claim. In addition, an inverse correlation between *Cyp4e3* and *TotA* is also intriguing because the relationship is consistently maintained in permethrin-treated flies and also in *Cyp4e3* knockdown flies [61]. Overall the available evidence points us to believe that the upregulation of *Cyp4e3* is universal and directly related to lipid accumulation or alteration in lipid metabolism.

### HFD mimics starvation related transcriptional change in the heads

This study also demonstrated HFD-induced upregulation of *takeout* gene in the heads of male and female flies (Fig 5A and 5B). A striking similarity of *takeout* upregulation in the heads is also reported as a result of starvation in the larvae [70]. Furthermore, in the adult flies the *takeout* mutant shows lower food intake post starvation [71]. Given that this gene is expressed in the head, cardia, crop, and antennae, and is known to regulate circadian rhythm and locomotor activity related to feeding behavior [70–72], we believe the possibility of this gene having a role in hyperphagic behavior. More importantly, since we know that both starvation and HFD induce hyperphagia in murines and humans [73, 74], the identification of gene with similar function to that of *takeout* in the mammals would be important.

## Conclusion

In the current study we used a comprehensive transcriptome analysis to explore both pervasive and sexually dimorphic alterations in the heads and in the bodies of flies with high dietary fat intake. There were distinct genes in the males and females that were activated by HFD but both converged to influence similar biological processes. Among the sexually dimorphic features, we show that the consumption of HFD leads to i) a female-centric enhancement of lipid metabolism and ii) a male-centric robust downregulation of genes related to stress response in the heads and downregulation of genes related to glycosidase activity in the bodies. It is also interesting to note that certain metabolic activities are sexually dimorphic even in the heads. However, the revelation that only males had stress response genes differentially expressed in their heads due to HFD is quite intriguing. The non-dimorphic observations include fat body specific genes differentially expressed, plausibly to regulate lipid homeostasis, and also a strong correlation between the *takeout* gene and hyperphagic behavior. Overall, our findings on transcriptional changes in flies to HFD can facilitate human studies investigating HFD-induced phenotype including obesity.

## Supporting information

S1 Fig Transcripts detected by RNA-Seq in the HFD versus RD treated males and females *D*. *melanogaster*. Scatterplots depicts the expression levels (TPM) of the entire transcriptome of the heads (A and B) and bodies (C and D) of males (A and C) and females (B and D) flies kept on HFD (Y-axis) versus RD (X-axis). *P*<0.001, red dots; 0.05<P<0.001, blue dots.
(TIF)

S2 Fig Differentially expressed genes in the body. A) Male body’s 130 differentially expressed genes at P<0.01. The 45 top ranked differentially expressed genes at P<0.001 are in the box. B) The female body’s 92 differentially expressed genes at P<0.01. The 22 top ranked differentially expressed genes at P<0.001 are in the box.
(TIF)

S1 Table Annotation Cluster analysis of the differentially expressed genes of males and females heads. (XLSX)

S2 Table Annotation Cluster analysis of the differentially expressed genes of males and females bodies. (XLSX)

S3 Table RT-PCR validation of the randomly selected gene from the top ranked differentially expressed genes detected from RNA-seq analysis in the head and body of males and females respectively. (XLSX)

## References

[1] Wang DD, Li Y, Chiuve SE, Stampfer MJ, Manson JE, Rimm EB, et al Association of Specific Dietary Fats With Total and Cause-Specific Mortality. JAMA Intern Med. 2016;176(8):1134–45. 10.1001/jamainternmed.2016.2417 27379574 PMC5123772

[2] Willett WC. Dietary fats and coronary heart disease. J Intern Med. 2012;272(1):13–24. 10.1111/j.1365-2796.2012.02553.x . 22583051

[3] Zong G, Li Y, Wanders AJ, Alssema M, Zock PL, Willett WC, et al Intake of individual saturated fatty acids and risk of coronary heart disease in US men and women: two prospective longitudinal cohort studies. BMJ. 2016;355:i5796 10.1136/bmj.i5796 www.icmje.org/coi_disclosure.pdf and declare: support from the National Institutes of Health for the submitted work; GZ is supported by a postdoctoral fellowship funded by Unilever R&D, Vlaardingen, Netherlands; AJW, MA, and PLZ are employees of Unilever R&D (Unilever is a producer of food consumer products); FBH has received research support from California Walnut Commission and Metagenics; no other relationships or activities that could appear to have influenced the submitted work. 27881409 PMC5121105

[4] Yoon PW, Bastian B, Anderson RN, Collins JL, Jaffe HW, Centers for Disease C, et al Potentially preventable deaths from the five leading causes of death—United States, 2008–2010. MMWR Morb Mortal Wkly Rep. 2014;63(17):369–74. 24785982 PMC4584887

[5] Della Vedova MC, Munoz MD, Santillan LD, Plateo-Pignatari MG, Germano MJ, Rinaldi Tosi ME, et al A Mouse Model of Diet-Induced Obesity Resembling Most Features of Human Metabolic Syndrome. Nutr Metab Insights. 2016;9:93–102. 10.4137/NMI.S32907 27980421 PMC5140012

[6] Phillips CM, Kesse-Guyot E, McManus R, Hercberg S, Lairon D, Planells R, et al High dietary saturated fat intake accentuates obesity risk associated with the fat mass and obesity-associated gene in adults. J Nutr. 2012;142(5):824–31. 10.3945/jn.111.153460 . 22457394

[7] Collaboration NCDRF. Worldwide trends in body-mass index, underweight, overweight, and obesity from 1975 to 2016: a pooled analysis of 2416 population-based measurement studies in 128.9 million children, adolescents, and adults. Lancet. 2017;390(10113):2627–42. 10.1016/S0140-6736(17)32129-3 . 29029897 PMC5735219

[8] Centers for Disease Control and Prevention. National Center for Chronic Disease Prevention and Health Promotion, Division of Nutrition, Physical Activity, and Obesity. Centers for Disease Control and Prevention. 2017.

[9] Flegal KM, Kruszon-Moran D, Carroll MD, Fryar CD, Ogden CL. Trends in Obesity Among Adults in the United States, 2005 to 2014. JAMA. 2016;315(21):2284–91. 10.1001/jama.2016.6458 . 27272580 PMC11197437

[10] Collaborators GBDO, Afshin A, Forouzanfar MH, Reitsma MB, Sur P, Estep K, et al Health Effects of Overweight and Obesity in 195 Countries over 25 Years. The New England journal of medicine. 2017;377(1):13–27. 10.1056/NEJMoa1614362 28604169 PMC5477817

[11] Eckel RH, Kahn SE, Ferrannini E, Goldfine AB, Nathan DM, Schwartz MW, et al Obesity and type 2 diabetes: what can be unified and what needs to be individualized? The Journal of clinical endocrinology and metabolism. 2011;96(6):1654–63. 10.1210/jc.2011-0585 21602457 PMC3206399

[12] Emerging Risk Factors C, Wormser D, Kaptoge S, Di Angelantonio E, Wood AM, Pennells L, et al Separate and combined associations of body-mass index and abdominal adiposity with cardiovascular disease: collaborative analysis of 58 prospective studies. Lancet. 2011;377(9771):1085–95. 10.1016/S0140-6736(11)60105-0 21397319 PMC3145074

[13] Pearson-Stuttard J, Zhou B, Kontis V, Bentham J, Gunter MJ, Ezzati M. Worldwide burden of cancer attributable to diabetes and high body-mass index: a comparative risk assessment. Lancet Diabetes Endocrinol. 2017 10.1016/S2213-8587(17)30366-2 . 29195904 PMC5805864

[14] Winocur G, Greenwood CE. Studies of the effects of high fat diets on cognitive function in a rat model. Neurobiol Aging. 2005;26 Suppl 1:46–9. 10.1016/j.neurobiolaging.2005.09.003 . 16219391

[15] Thaler JP, Schwartz MW. Minireview: Inflammation and obesity pathogenesis: the hypothalamus heats up. Endocrinology. 2010;151(9):4109–15. 10.1210/en.2010-0336 20573720 PMC2940486

[16] Jayaraman A, Lent-Schochet D, Pike CJ. Diet-induced obesity and low testosterone increase neuroinflammation and impair neural function. J Neuroinflammation. 2014;11:162 10.1186/s12974-014-0162-y ; PubMed Central PMCID: PMCPMC4190446. 25224590 PMC4190446

[17] Sellbom KS, Gunstad J. Cognitive function and decline in obesity. J Alzheimers Dis. 2012;30 Suppl 2:S89–95. 10.3233/JAD-2011-111073 . 22258511

[18] Kalmijn S. Fatty acid intake and the risk of dementia and cognitive decline: a review of clinical and epidemiological studies. J Nutr Health Aging. 2000;4(4):202–7. . 11115801

[19] Moser VA, Pike CJ. Obesity and sex interact in the regulation of Alzheimer's disease. Neurosci Biobehav Rev. 2016;67:102–18. 10.1016/j.neubiorev.2015.08.021 26708713 PMC4912955

[20] Williamson R, McNeilly A, Sutherland C. Insulin resistance in the brain: an old-age or new-age problem? Biochemical pharmacology. 2012;84(6):737–45. 10.1016/j.bcp.2012.05.007 . 22634336

[21] Langdon KD, Clarke J, Corbett D. Long-term exposure to high fat diet is bad for your brain: exacerbation of focal ischemic brain injury. Neuroscience. 2011;182:82–7. 10.1016/j.neuroscience.2011.03.028 . 21435380

[22] Freeman LR, Haley-Zitlin V, Rosenberger DS, Granholm AC. Damaging effects of a high-fat diet to the brain and cognition: a review of proposed mechanisms. Nutr Neurosci. 2014;17(6):241–51. 10.1179/1476830513Y.0000000092 24192577 PMC4074256

[23] Pistell PJ, Morrison CD, Gupta S, Knight AG, Keller JN, Ingram DK, et al Cognitive impairment following high fat diet consumption is associated with brain inflammation. J Neuroimmunol. 2010;219(1–2):25–32. 10.1016/j.jneuroim.2009.11.010 20004026 PMC2823983

[24] Sharma S, Fulton S. Diet-induced obesity promotes depressive-like behaviour that is associated with neural adaptations in brain reward circuitry. Int J Obes (Lond). 2013;37(3):382–9. 10.1038/ijo.2012.48 . 22508336

[25] Valladolid-Acebes I, Fole A, Martin M, Morales L, Cano MV, Ruiz-Gayo M, et al Spatial memory impairment and changes in hippocampal morphology are triggered by high-fat diets in adolescent mice. Is there a role of leptin? Neurobiol Learn Mem. 2013;106:18–25. 10.1016/j.nlm.2013.06.012 . 23820496

[26] Yang JL, Liu X, Jiang H, Pan F, Ho CS, Ho RC. The Effects of High-fat-diet Combined with Chronic Unpredictable Mild Stress on Depression-like Behavior and Leptin/LepRb in Male Rats. Sci Rep. 2016;6:35239 10.1038/srep35239 27739518 PMC5064321

[27] Le Foll C, Levin BE. Fatty acid-induced astrocyte ketone production and the control of food intake. Am J Physiol Regul Integr Comp Physiol. 2016;310(11):R1186–92. 10.1152/ajpregu.00113.2016 27122369 PMC4935491

[28] Wang GJ, Volkow ND, Telang F, Jayne M, Ma Y, Pradhan K, et al Evidence of gender differences in the ability to inhibit brain activation elicited by food stimulation. Proceedings of the National Academy of Sciences of the United States of America. 2009;106(4):1249–54. 10.1073/pnas.0807423106 19164587 PMC2633545

[29] Mueller K, Anwander A, Moller HE, Horstmann A, Lepsien J, Busse F, et al Sex-dependent influences of obesity on cerebral white matter investigated by diffusion-tensor imaging. PloS one. 2011;6(4):e18544 10.1371/journal.pone.0018544 ; PubMed Central PMCID: PMCPMC3073967. 21494606 PMC3073967

[30] Udo T, Grilo CM, McKee SA. Gender differences in the impact of stressful life events on changes in body mass index. Prev Med. 2014;69:49–53. 10.1016/j.ypmed.2014.08.036 ; PubMed Central PMCID: PMCPMC4312235. 25204986 PMC4312235

[31] Bekhbat M, Neigh GN. Sex differences in the neuro-immune consequences of stress: Focus on depression and anxiety. Brain Behav Immun. 2018;67:1–12. 10.1016/j.bbi.2017.02.006 28216088 PMC5559342

[32] Heinrichsen ET, Haddad GG. Role of high-fat diet in stress response of Drosophila. PloS one. 2012;7(8):e42587 10.1371/journal.pone.0042587 22870336 PMC3411628

[33] Heinrichsen ET, Zhang H, Robinson JE, Ngo J, Diop S, Bodmer R, et al Metabolic and transcriptional response to a high-fat diet in Drosophila melanogaster. Molecular metabolism. 2014;3(1):42–54. Epub 2014/02/26. 10.1016/j.molmet.2013.10.003 24567903 PMC3929909

[34] Birse RT, Choi J, Reardon K, Rodriguez J, Graham S, Diop S, et al High-fat-diet-induced obesity and heart dysfunction are regulated by the TOR pathway in Drosophila. Cell Metab. 2010;12(5):533–44. 10.1016/j.cmet.2010.09.014 21035763 PMC3026640

[35] Prussing K, Voigt A, Schulz JB. Drosophila melanogaster as a model organism for Alzheimer's disease. Mol Neurodegener. 2013;8:35 10.1186/1750-1326-8-35 ; PubMed Central PMCID: PMCPMC4222597. 24267573 PMC4222597

[36] Jeibmann A, Paulus W. Drosophila melanogaster as a model organism of brain diseases. Int J Mol Sci. 2009;10(2):407–40. 10.3390/ijms10020407 19333415 PMC2660653

[37] Bier E., Drosophila the golden bug, emerges as a tool for human genetics. Nat Rev Genet. 2005;6(1):9–23. 10.1038/nrg1503 . 15630418

[38] Ayroles JF, Carbone MA, Stone EA, Jordan KW, Lyman RF, Magwire MM, et al Systems genetics of complex traits in Drosophila melanogaster. Nature genetics. 2009;41(3):299–307. 10.1038/ng.332 19234471 PMC2752214

[39] Kishita Y, Tsuda M, Aigaki T. Impaired fatty acid oxidation in a Drosophila model of mitochondrial trifunctional protein (MTP) deficiency. Biochemical and biophysical research communications. 2012;419(2):344–9. 10.1016/j.bbrc.2012.02.026 . 22342726

[40] Sellin J, Wingen C, Gosejacob D, Senyilmaz D, Hanschke L, Buttner S, et al Dietary rescue of lipotoxicity-induced mitochondrial damage in Peroxin19 mutants. PLoS Biol. 2018;16(6):e2004893 10.1371/journal.pbio.2004893 29920513 PMC6025876

[41] Ganz M, Csak T, Szabo G. High fat diet feeding results in gender specific steatohepatitis and inflammasome activation. World J Gastroenterol. 2014;20(26):8525–34. 10.3748/wjg.v20.i26.8525 25024607 PMC4093702

[42] Benz V, Bloch M, Wardat S, Bohm C, Maurer L, Mahmoodzadeh S, et al Sexual dimorphic regulation of body weight dynamics and adipose tissue lipolysis. PloS one. 2012;7(5):e37794 10.1371/journal.pone.0037794 ; PubMed Central PMCID: PMCPMC3360591. 22662224 PMC3360591

[43] Gupte M, Thatcher SE, Boustany-Kari CM, Shoemaker R, Yiannikouris F, Zhang X, et al Angiotensin converting enzyme 2 contributes to sex differences in the development of obesity hypertension in C57BL/6 mice. Arterioscler Thromb Vasc Biol. 2012;32(6):1392–9. 10.1161/ATVBAHA.112.248559 22460555 PMC3355213

[44] Horton TJ, Dow S, Armstrong M, Donahoo WT. Greater systemic lipolysis in women compared with men during moderate-dose infusion of epinephrine and/or norepinephrine. J Appl Physiol (1985). 2009;107(1):200–10. 10.1152/japplphysiol.90812.2008 19407251 PMC2711790

[45] Ekengren S, Tryselius Y, Dushay MS, Liu G, Steiner H, Hultmark D. A humoral stress response in Drosophila. Current biology: CB. 2001;11(18):1479 . 11566110 10.1016/s0960-9822(01)00452-3

[46] Seong KM, Coates BS, Sun W, Clark JM, Pittendrigh BR. Changes in Neuronal Signaling and Cell Stress Response Pathways are Associated with a Multigenic Response of Drosophila melanogaster to DDT Selection. Genome Biol Evol. 2017;9(12):3356–72. 10.1093/gbe/evx252 29211847 PMC5737697

[47] Moskalev A, Shaposhnikov M, Proshkina E, Belyi A, Fedintsev A, Zhikrivetskaya S, et al The influence of pro-longevity gene Gclc overexpression on the age-dependent changes in Drosophila transcriptome and biological functions. BMC Genomics. 2016;17(Suppl 14):1046 10.1186/s12864-016-3356-0 28105938 PMC5249042

[48] Oakes ND, Cooney GJ, Camilleri S, Chisholm DJ, Kraegen EW. Mechanisms of liver and muscle insulin resistance induced by chronic high-fat feeding. Diabetes. 1997;46(11):1768–74. . 9356024 10.2337/diab.46.11.1768

[49] Cattaneo F, Pasini ME, Perotti ME. Glycosidases are present on the surface of Drosophila melanogaster spermatozoa. Mol Reprod Dev. 1997;48(2):276–81. 10.1002/(SICI)1098-2795(199710)48:2<276::AID-MRD16>3.0.CO;2-W . 9291478

[50] Mu Y, Yan WJ, Yin TL, Zhang Y, Li J, Yang J. Diet-induced obesity impairs spermatogenesis: a potential role for autophagy. Sci Rep. 2017;7:43475 10.1038/srep43475 ; PubMed Central PMCID: PMCPMC5343591. 28276438 PMC5343591

[51] Andersen JM, Herning H, Aschim EL, Hjelmesaeth J, Mala T, Hanevik HI, et al Body Mass Index Is Associated with Impaired Semen Characteristics and Reduced Levels of Anti-Mullerian Hormone across a Wide Weight Range. PloS one. 2015;10(6):e0130210 10.1371/journal.pone.0130210 26067627 PMC4466334

[52] Kuhnlein RP. Thematic review series: Lipid droplet synthesis and metabolism: from yeast to man. Lipid droplet-based storage fat metabolism in Drosophila. J Lipid Res. 2012;53(8):1430–6. 10.1194/jlr.R024299 22566574 PMC3540836

[53] Benes H, Spivey DW, Miles J, Neal K, Edmondson RG. Fat-body-specific expression of the Drosophila Lsp-2 gene. SAAS Bull Biochem Biotechnol. 1990;3:129–33. . 1369981

[54] Telfer WH, Kunkel JG. The function and evolution of insect storage hexamers. Annu Rev Entomol. 1991;36:205–28. 10.1146/annurev.en.36.010191.001225 . 2006868

[55] Jowett T, Postlethwait JH. Hormonal regulation of synthesis of yolk proteins and a larval serum protein (LSP2) in Drosophila. Nature. 1981;292(5824):633–5. . 7254357 10.1038/292633a0

[56] Bownes M, Blair M, Kozma R, Dempster M. 20-hydroxyecdysone stimulates tissue-specific yolk-protein gene transcription in both male and female Drosophila. J Embryol Exp Morphol. 1983;78:249–68. . 6198419

[57] Wang S, Liu S, Liu H, Wang J, Zhou S, Jiang RJ, et al 20-hydroxyecdysone reduces insect food consumption resulting in fat body lipolysis during molting and pupation. J Mol Cell Biol. 2010;2(3):128–38. 10.1093/jmcb/mjq006 . 20430856

[58] Sieber MH, Thummel CS. The DHR96 nuclear receptor controls triacylglycerol homeostasis in Drosophila. Cell Metab. 2009;10(6):481–90. 10.1016/j.cmet.2009.10.010 19945405 PMC2803078

[59] Ellis LL, Carney GE. Mating alters gene expression patterns in Drosophila melanogaster male heads. BMC Genomics. 2010;11:558 10.1186/1471-2164-11-558 20937114 PMC3091707

[60] Obata F, Kuranaga E, Tomioka K, Ming M, Takeishi A, Chen CH, et al Necrosis-driven systemic immune response alters SAM metabolism through the FOXO-GNMT axis. Cell Rep. 2014;7(3):821–33. 10.1016/j.celrep.2014.03.046 . 24746817

[61] Terhzaz S, Cabrero P, Brinzer RA, Halberg KA, Dow JA, Davies SA. A novel role of Drosophila cytochrome P450-4e3 in permethrin insecticide tolerance. Insect Biochem Mol Biol. 2015;67:38–46. 10.1016/j.ibmb.2015.06.002 26073628 PMC4673087

[62] Bauer J, Antosh M, Chang C, Schorl C, Kolli S, Neretti N, et al Comparative transcriptional profiling identifies takeout as a gene that regulates life span. Aging (Albany NY). 2010;2(5):298–310. 10.18632/aging.100146 20519778 PMC2898020

[63] Chung H, Sztal T, Pasricha S, Sridhar M, Batterham P, Daborn PJ. Characterization of Drosophila melanogaster cytochrome P450 genes. Proceedings of the National Academy of Sciences of the United States of America. 2009;106(14):5731–6. 10.1073/pnas.0812141106 19289821 PMC2667016

[64] Logan-Garbisch T, Bortolazzo A, Luu P, Ford A, Do D, Khodabakhshi P, et al Developmental ethanol exposure leads to dysregulation of lipid metabolism and oxidative stress in Drosophila. G3 (Bethesda). 2014;5(1):49–59. 10.1534/g3.114.015040 25387828 PMC4291469

[65] Musselman LP, Fink JL, Ramachandran PV, Patterson BW, Okunade AL, Maier E, et al Role of fat body lipogenesis in protection against the effects of caloric overload in Drosophila. The Journal of biological chemistry. 2013;288(12):8028–42. 10.1074/jbc.M112.371047 23355467 PMC3605622

[66] Musselman LP, Fink JL, Narzinski K, Ramachandran PV, Hathiramani SS, Cagan RL, et al A high-sugar diet produces obesity and insulin resistance in wild-type Drosophila. Disease models & mechanisms. 2011;4(6):842–9. 10.1242/dmm.007948 ; PubMed Central PMCID: PMCPMC3209653. 21719444 PMC3209653

[67] Xiao X, Qi W, Clark JM, Park Y. Permethrin potentiates adipogenesis via intracellular calcium and endoplasmic reticulum stress-mediated mechanisms in 3T3-L1 adipocytes. Food Chem Toxicol. 2017;109(Pt 1):123–9. 10.1016/j.fct.2017.08.049 . 28870683

[68] Lockhart CM, Nakano M, Rettie AE, Kelly EJ. Generation and characterization of a murine model of Bietti crystalline dystrophy. Invest Ophthalmol Vis Sci. 2014;55(9):5572–81. 10.1167/iovs.13-13717 25118264 PMC4160072

[69] Li S, Li Q, Kong Y, Wu S, Cui Q, Zhang M, et al Specific regulation of thermosensitive lipid droplet fusion by a nuclear hormone receptor pathway. Proceedings of the National Academy of Sciences of the United States of America. 2017;114(33):8841–6. 10.1073/pnas.1704277114 28760992 PMC5565433

[70] Sarov-Blat L, So WV, Liu L, Rosbash M. The Drosophila takeout gene is a novel molecular link between circadian rhythms and feeding behavior. Cell. 2000;101(6):647–56. . 10892651 10.1016/s0092-8674(00)80876-4

[71] Meunier N, Belgacem YH, Martin JR. Regulation of feeding behaviour and locomotor activity by takeout in Drosophila. J Exp Biol. 2007;210(Pt 8):1424–34. 10.1242/jeb.02755 . 17401125

[72] So WV, Sarov-Blat L, Kotarski CK, McDonald MJ, Allada R, Rosbash M. takeout, a novel Drosophila gene under circadian clock transcriptional regulation. Molecular and cellular biology. 2000;20(18):6935–44. 10958689 10.1128/mcb.20.18.6935-6944.2000 PMC88769

[73] Warwick ZS, Weingarten HP. Determinants of high-fat diet hyperphagia: experimental dissection of orosensory and postingestive effects. The American journal of physiology. 1995;269(1 Pt 2):R30–7. 10.1152/ajpregu.1995.269.1.R30 . 7631900

[74] Dulloo AG, Jacquet J, Girardier L. Poststarvation hyperphagia and body fat overshooting in humans: a role for feedback signals from lean and fat tissues. Am J Clin Nutr. 1997;65(3):717–23. 10.1093/ajcn/65.3.717 . 9062520

