# Supplementary figures and images for: High fat diet induces sex-specific differential gene expression in Drosophila melanogaster
Source: PLoS One. 2019 Mar 12;14(3):e0213474. doi: 10.1371/journal.pone.0213474 (PMC6413938; doi:10.1371/journal.pone.0213474)

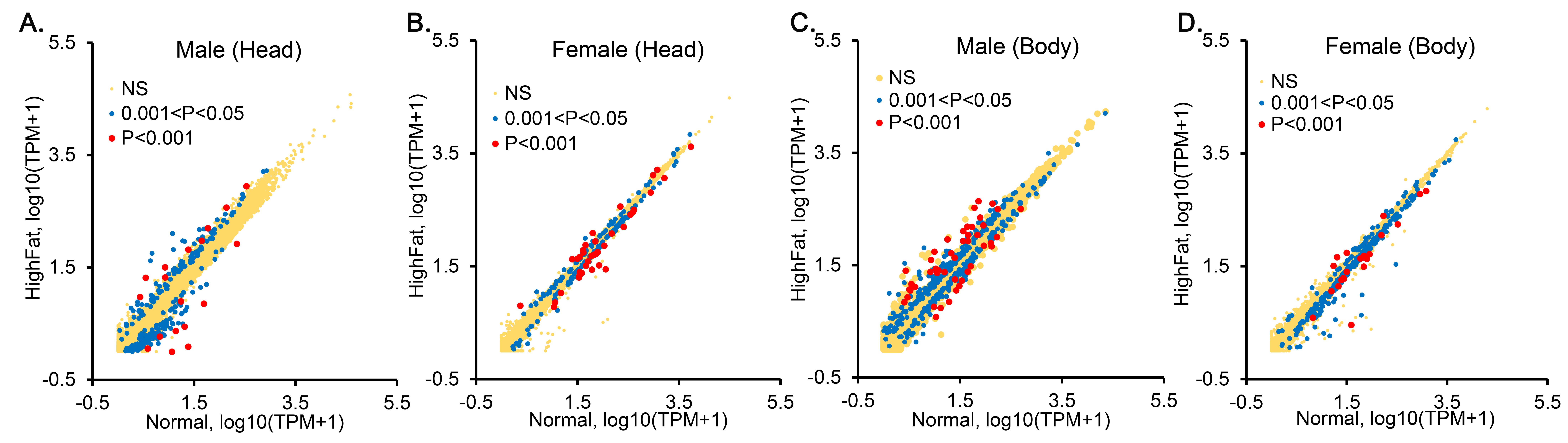

Supplement: S1 Fig — Scatterplots depicts the expression levels (TPM) of the entire transcriptome of the heads (A and B) and bodies (C and D) of males (A and C) and females (B and D) flies kept on HFD (Y-axis) versus RD (X-axis). P<0.001, red dots; 0.05<P<0.001, blue dots. (TIF) [file pone.0213474.s001.tif]

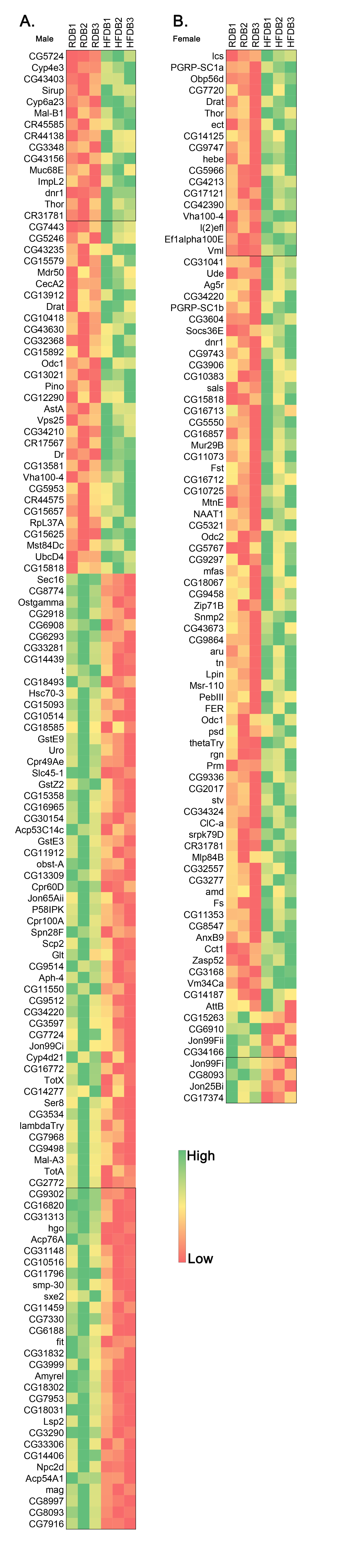

Supplement: S2 Fig — A) Male body’s 130 differentially expressed genes at P<0.01. The 45 top ranked differentially expressed genes at P<0.001 are in the box. B) The female body’s 92 differentially expressed genes at P<0.01. The 22 top ranked differentially expressed genes at P<0.001 are in the box. (TIF) [file pone.0213474.s002.tif]
